# Adherence with isoniazid for prevention of tuberculosis among HIV-infected adults in South Africa

**DOI:** 10.1186/1471-2334-6-97

**Published:** 2006-06-13

**Authors:** Tom A Szakacs, Douglas Wilson, D William Cameron, Michael Clark, Paul Kocheleff, F James Muller, Anne E McCarthy

**Affiliations:** 1 Division of Infectious Diseases, University of Ottawa at The Ottawa Hospital, 501 Smyth Rd., K1H 8L6, Ottawa, Canada; 2 Department of Medicine, Pietermaritzburg Hospital Complex, Greys Hospital, 3200 Townbush Rd., Pietermaritzburg, South Africa

## Abstract

**Background:**

Tuberculosis (TB) is the most common opportunistic infection in HIV-infected adults in developing countries. Isoniazid (INH) is recommended for treatment of latent TB infection, however non-adherence is common. The purpose of this study was to apply in-house prepared isoniazid (INH) urine test strips in a clinical setting, and identify predictors of positive test results in an adherence questionnaire in HIV-infected adults taking INH for prevention of TB.

**Methods:**

Cross-sectional study of adherence using a questionnaire and urine test strips for detection of INH metabolites at two hospitals in Pietermaritzburg, South Africa. Participants were aged at least 18 years, HIV positive, and receiving INH for prevention of tuberculosis disease. Univariate and multivariate analyses are used to identify factors relevant to adherence.

**Results:**

301 consecutive patients were recruited. 28% of participants had negative urine tests. 32 (37.2%, 95% CI25.4, 45.0) of the 86 patients who received INH from peripheral pharmacies said the pharmacy had run out of INH at some time, compared with central hospital pharmacies (p = 0.0001). In univariate analysis, a negative test was associated with self-reported missed INH doses (p = 0.043). Each 12-hour increment since last reported dose increased the likelihood of a negative test by 34% (p = 0.0007). Belief in INH safety was associated with a positive test (p = 0.021). In multivariate analysis, patients who believed INH is important for prevention of TB disease were more likely to be negative (p = 0.0086).

**Conclusion:**

Adequate drug availability at peripheral pharmacies remains an important intervention for TB prevention. Key questions may identify potentially non-adherent patients. In-house prepared urine tests strips are an effective and cheap method of objectively assessing INH adherence, and could be used an important tool in TB control programs.

## Background

Tuberculosis (TB) remains a major global epidemic despite widespread awareness and effective prevention and therapy. Human immunodeficiency virus (HIV) infection has had a devastating effect on the TB epidemic. In South Africa an estimated two million people are co-infected, as are 60% of all patients with newly diagnosed TB [1].

Accordingly, the World Health Organization and American Thoracic Society now recommend INH for treatment of latent TB infection (LTBI) in HIV-infected patients who are TST positive [2,3]. If skin testing is not feasible, INH is still recommended in regions with high TB prevalence [2].

Studies have shown rates of non-adherence to be typically 8–33% [4-13]. One objective measure of adherence is a biochemical test called the Arkansas method, where a chemical reaction with urinary INH metabolites produces a visible blue colour change [4,14,15]. This method has a high sensitivity (>99%) and specificity (>96%) [4,7,15]. While commercial test strips are not readily available in developing countries, they can be reproduced in-house at very little cost, without compromising quality [6,15]. Compared to commercial test strips, in-house prepared strips have a sensitivity and specificity of 99.5% and 96.4% respectively [6]. Their low cost, ease of use, and accuracy make them an ideal evaluative component of TB control programs.

The aim of our study was to apply in-house prepared INH urine test strips in a clinical setting in HIV-infected adults taking INH for prevention of TB, and to identify predictors of positive urine test results in an adherence questionnaire at two South African hospitals.

## Methods

### Setting

The study sample consisted of consecutive outpatients from HIV clinics at two hospitals in Pietermaritzburg, South Africa. Hospital A provides tertiary care and is located in an affluent suburban area; attendees pay US $3 for a consultation or medication. Hospital B is a district hospital in an impoverished suburban area, with limited access to specialist care and significantly less modern facilities. Patients may attend either hospital's HIV clinic; the same physicians staff clinics at both sites. Few patients were on antiretroviral therapy at the time of the study as the anti-retroviral rollout had not yet occurred.

Patients fill their INH prescription at the hospital pharmacy or at community pharmacies that are funded by the provincial government.

Regular clinic attendees were asked to participate in the study if they were 18 years or greater, HIV infected and prescribed INH (300 mg daily) with pyridoxine for prevention of TB. Clinic policy was not to perform TST prior to initiating therapy, and to continue treatment indefinitely. Patients receiving INH as part of multi-drug therapy for active TB and those with frank hematuria were excluded from the study. All participants gave written informed consent. The questionnaire and urine test were performed once in each patient with no follow up testing.

### Procedures

Consecutive patients who met entry criteria were invited to participate by a study nurse. For those accepting, a nurse administered the questionnaire and performed the urine test prior to their appointment with the physician. One question asked participants to explain why they were taking INH. A correct response was if they indicated the INH was either to prevent or treat TB.

The preliminary questionnaire was developed from review of previous studies of factors affecting adherence [6-9,13,16-20]. Additionally relevant questions were subsequently added after review by all the authors. The questionnaire was available in English or a translated Zulu form depending on patient preference.

Test strips were prepared via the description by Kilburn et al [15]. Filter paper [21] (cut into strips 6 × 0.8 cm) was marked at one end with a graphite pencil. The following stock solutions were prepared: 5% barbituric acid (pH adjusted to 5.2 using 50% sodium hydroxide), 60% potassium thiocyanate in 8% citric acid, and 50% chloramine T. Distilled water was the base medium in all solutions. The barbituric acid and chloramine T solutions were warmed prior to spotting to aid in solubilization. Each strip was spotted with 25 μl in three separate bands, with chloramine T and barbituric acid at the ends and potassium thiocyanate in the middle. The barbituric acid was spotted over the end of the strip previously marked; the strips were then air-dried and stored. In the present study, a single individual (TAS) made 500 test strips in four hours. There were no special laboratory needs; only the chemicals and standard lab equipment were required. Each strip cost less than 1 US cent to prepare.

Midstream urine was collected in a standard urine bottle. To detect INH, 0.5 ml of patient urine was pipetted from the urine bottle into a test tube, a test strip was added and a stopper applied. The strips were placed with the barbituric acid end downward. After one hour the urine and test strip were read. Patients' urine was deemed positive (INH metabolite present) if the urine or test strip turned a blue-green to dark blue colour, and negative (no INH metabolite present) if the urine and test strip remained yellow. Urine testing was performed just prior to the questionnaire. The questionnaire and urine test were completed on each participant once, with no further follow up testing. For the purpose of this study, patients were deemed to be adherent or non-adherent based on the result of the urine test. To ensure that the test strips were grossly effective, prior to initiating patient recruitment, urine test strips were pilot tested by 5 volunteers from the Department of Medicine at Hospital A. Each volunteer took INH 300 mg once, and had urine samples checked at time 0, 24, 36 and 72 hours. The color of the urine and test strip was recorded.

This study is in compliance with the Helsinki Declaration and received ethical approval from the Ottawa Hospital Regional Ethics Board and the Research Ethics Committee, Nelson R. Mandela School of Medicine, University of KwaZulu Natal.

### Statistical analysis

Descriptive statistics were generated for the overall patient population, including means and standard deviations for continuous variables, and proportions and 95% confidence limits for categorical variables. These statistics were then calculated for each hospital. Means and proportions were compared using t-tests and chi-square testing, respectively. Exploratory analyses were conducted on variables relevant to medication availability and provision in dispensing pharmacies. Associations between variables and urine test results (positive or negative) were assessed with univariate and multivariate logistic regression analyses. In multivariate analyses, a forward selection procedure was used, beginning with variables that were found to be significant predictors in univariate analyses. Variables were entered into the model if p < 0.15, and taken out if p > 0.15. The model's overall fit to the data was assessed with Hosmer and Lemeshow goodness-of-fit testing. All of the above analyses were conducted using SAS 8 statistical software (SAS Institute, Cary, NC, USA).

## Results

During the urine test strip pilot testing by 5 volunteers, all samples were positive at 24 hours, and became negative at 72 hours, consistent with the expected pattern.^14 ^The individual results of the tests are shown in Additional file 1.

Between February and May 2004, 304 consecutive patients meeting entry criteria were identified. Consent was obtained from 301 of these patients, while 3 patients refused to participate. All of the questionnaire items and their responses are shown in Additional file 2. The full English version of the questionnaire is shown in Additional file 3.

The mean age of study participants was 35, with 76.7% being female. Each household had an average of 6.3 people and a total monthly income of US $238. The mean duration since HIV diagnosis was 33.3 months (SD 26) and mean time on INH therapy was 18.7 months (SD 17). On average, participants had told 3.0 people they were taking INH and 3.1 people that they have HIV, while a substantial portion (20.7% and 17.3% respectively) had not told anyone. A total of 18 patients (6%) were on anti-retrovirals.

Overall, 28.0% of urine tests were negative. There was no significant difference in urine results between the two hospitals; however, there were a number of qualitative differences (see Additional file 2). In particular, there was considerable variation in where study participants get their INH. At Hospital A only 4 patients (4.0%, 95% CI 0.2, 7.9) attended peripheral pharmacies, while the remainder attended the hospital pharmacy. In contrast, at Hospital B, 86 patients (42.8%, 95% CI 35.9, 49.6) attended peripheral pharmacies. For patients getting their INH at Hospital A, 42.1% (95% CI 32.2, 52.0) said they sometimes could not afford to go to the pharmacy. This occurred in only 3.5% (95% CI 0.001, 0.0069) attending the Hospital B pharmacy, and 1.1% attending peripheral pharmacies (χ^2 ^= 80.3, p = 0.0001). The proportion of patients who ran out of INH between hospital visits was 7.3%, 19.3%, and 26.4%, among patients receiving INH from Hospital A, Hospital B, and peripheral pharmacies, respectively (χ^2 ^= 12.1, p = 0.0024). Only 1 patient at Hospital B and none at Hospital A reported their pharmacy had previously run out of INH. However, this occurred for 32 patients (37.2%, 95% CI 25.4, 45.0) attending peripheral pharmacies (χ^2 ^= 78.3, p = 0.0001). Participants attended 25 different peripheral pharmacies; 8 of these serviced 73 of the total 86 patients (80.2%). No particular pharmacy was statistically associated with urine test results; however 41.1% of patients attending these 8 pharmacies said it had run out of medications at some time. At the most frequented pharmacy, 10 of 18 patients (55.6%) reported this had occurred.

**Figure 1:**
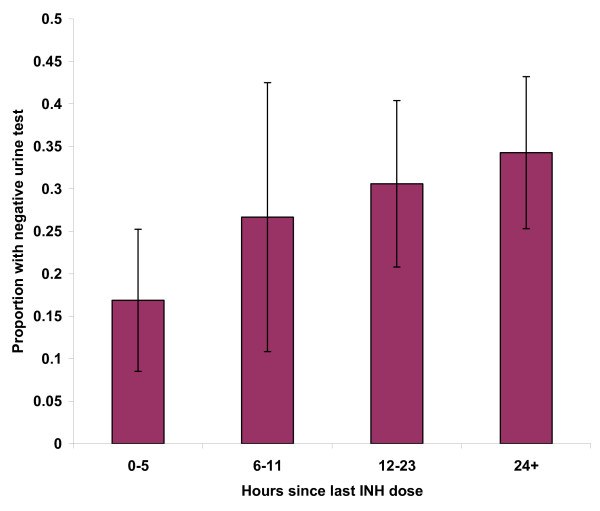
Urine result based on time since last self-reported INH dose (95% CI shown in bars).

In univariate logistic regression analysis, three factors were found to have a statistically significant impact on urine test results (Table 1). Longer time since last self-reported INH dose showed a stepwise increase in probability of having a negative urine test (OR 1.33; 95% CI, 1.13 to 1.57; p = 0.0007). This relationship is shown in Figure 1. Every 12-hour increase in reported time resulted in a 34% (95% CI 13%, 57%) increase in the probability of a negative urine test. Those who believed the INH was safe were less likely to have a negative urine test (OR 0.47; 95% CI, 0.25 to 0.90; p = 0.021). There was also a non-significant trend towards increased compliance in those who believed INH was not safe (OR 0.71; 95% CI, 0.29 to 1.75; p = 0.45). Finally, patients who reported they sometimes forget to take the INH were more likely to have negative tests (OR 2.0; 95% CI, 1.02–3.99; p = 0.043).

**Table 1:** Univariate logistic regression analyses of study variables showing a significant association with a negative urine test result

**Variable**	**OR (95% CI)**	**p value**
How long since last INH dose? (12-hour increments)?	1.33 (1.13, 1.57)	0.0007
Statement: INH is dangerous to your health		
Strongly agree/agree	0.71 (0.29, 1.75)	0.45
Disagree/strongly disagree	0.47 (0.25, 0.90)	0.021
Don't know	1.00	
Do you ever forget to take INH?	2.0 (1.02, 3.99)	0.043

Time since last reported INH dose and probability of a negative test remained unchanged in all multivariate analyses (Table 2). As well, belief that INH is safe continued to decrease the likelihood of a negative urine test (p = 0.0098). Overall, only nine patients in the study answered that their chance of getting TB without INH were average or below average. Those who felt their chances of getting sick from TB without INH were above average or high were more likely to have negative urine results, when compared to those who didn't know (OR 3.00; 95% CI, 1.32 to 6.79; p = 0.0086). The variable of patient forgetfulness in taking the INH was not found to be significant in the multivariate analysis.

**Table 2:** Multivariate logistic regression model of study variables predicting a negative urine test result

**Variable**	**Parameter estimate**	**OR (95% CI)**	**p value**
Intercept	-1.84		0.0064
How long since last INH dose? (12-hour increments)?	0.30	1.34 (1.13, 1.59)	0.0007
Statement: INH is dangerous to your health			
Strongly agree/agree	-0.48	0.62 (0.21, 1.81)	0.38
Disagree/strongly disagree	-1.08	0.34 (0.15, 0.77)	0.0098
Don't know	0	1.00	
Statement: Without INH, your chance of getting sick from TB is:			
High/above average	1.10	3.00 (1.32, 6.79)	0.0086
Average/below average	0.96	2.61 (0.49, 13.8)	0.26
Don't know	0	1.00	

Goodness-of-fit statistic = 4.28 (p = 0.83)

## Discussion

Urine testing measured the overall rate of non-adherence at 28%. This is similar to other studies that have shown rates varying between 8–33% [4-13]. However, there are few consistent predictors of adherence between studies. Some show that homelessness, alcoholism, adherence counselling, race, prior BCG, sex, and age had significant influences [7,8,13,16-18]. Others point to social factors [19,20], and still others have found no correlative factors [5,6]. In the present study a number of predictive factors were found, including the self reported time since last INH dose. When asked how frequently they take the INH, 95.3% of participants said daily, reflecting their understanding that INH should be taken daily. If broadly asked about adherence in judgmental or threatening contexts, one may not be straightforward. However, a less threatening question, such as time since last dose is valid in comparison with urine testing. Use of 12-hour time increments provides an easy estimate of the probability of a negative urine test. This confirms the same relationship that was found in a study of LTBI by Perry et al [8].

Patients who report they sometimes forget to take their medication were more likely to have a negative urine test. This was previously identified as a predictor in another study of patients on INH for LTBI [9]. There, 33% admitted to forgetting to take the INH, while this occurred in 14% of our study.

Those who expressed any opinion as to the safety of INH were less likely to test negative (i.e., more likely adherent to therapy). Participants who disagreed that INH was a danger to their health were significantly less likely to test negative in univariate and multivariate analyses, than those who stated they did not know. However, it is interesting that those who agreed that INH is dangerous were also less likely to test negative, without statistical significance. This may reflect the small number of respondents who "strongly agreed" or "agreed" with the statement (1.7% and 8.7% of respondents, respectively), or may represent lack of understanding of the question.

Patients were on INH for an average of 18.7 months, longer than the typical 6–12 month course [2,3]. In Pietermaritzburg, longer therapy is used as primary and secondary prophylaxis to prevent new infection from ongoing exposure. Although this contrasts to current international guidelines, there are studies supporting this approach [22-24]. One study in South Africa examined indefinite secondary therapy in HIV-infected patients with prior active TB and found a 55% reduction in the incidence of TB recurrence [22].

Unlike other studies where INH side effects impacted adherence [7,13,16] we did not find this relationship. In fact, only 6% reported this, compared to up to 74% in other studies [13]. While this may be due to selection of patients that had been able to tolerate INH for a prolonged time, univariate and multivariate testing did not find that duration of treatment affected adherence.

In multivariate analysis, those believing they have a high or above average chance of getting active TB without INH were more likely non-adherent. While this seems paradoxical, it might be explained by social factors. Patients may perceive stigma or conceal their condition and its treatment, which could interfere with adherence. Alternatively, knowledge of the danger of TB may motivate them to share or sell their INH to others who are also at risk. As this relationship is counter-intuitive it merits further study.

To our knowledge, ours is the first study to identify location of drug supply as a factor in adherence. An alarming number of peripheral pharmacies are reported to run out of medications, which may impair overall adherence. Although pharmacy location did not significantly impact urine test results, the study was not designed to detect this difference. Regardless, this identification of impaired access to medicines may represent an important barrier to overall adherence.

In areas where patients attend peripheral pharmacies, TB programs should include resources to routinely audit and address supply problems. Alternatively, it may be necessary to subsidize or facilitate transportation to hospital pharmacies. These conclusions have additional relevance in the context of the South African antiretroviral rollout, which ultimately will be delivered by primary health care facilities.

To our knowledge, only the present study and that by Meissner et al [6] have applied in-house prepared test strips. However, in our study we identified qualitative issues not described previously. Only when the barbituric acid end was placed in the urine would the reaction proceed appropriately, hence that end was marked with a pencil. Otherwise, strips with the chloramine T end downward were uniformly negative. Also, previous studies say results were available within 10–15 minutes [6,15]. While this was true for the majority, we found a small portion of results remained ambiguous, and when we extended the evaluation time to one hour some samples then became more clearly positive.

Some factors may have an impact on use of the urine test. The rate of INH metabolism is affected by liver acetylator status, with some patients exhibiting rapid drug metabolism. No study has attempted to address the impact of this status on the urine test results. Also, there are conflicting reports on impaired INH absorption in HIV and TB co-infection [25-27]. Lastly, it may seem attractive to extrapolate the urine test for assessing compliance in patients with active tuberculosis receiving multi-drug therapy. However, no study has formally examined the biochemical impact that other anti-tuberculosis medications may have on urine testing for INH metabolites.

Although the urine test is a single point measurement and does not necessarily reflect overall adherence, its biochemical characteristics make it a useful predictor. 48 hours after the last dose 76% of patients will have a positive result, while after 72 hours only 6% will remain positive [14]. In fact, one study showed that urine testing is an accurate estimate of overall adherence when compared to electronic monitoring of pill-bottle lid opening [28]. Another study performed random urine testing between scheduled appointments and found similar rates of adherence between visits as at appointments [9]. So, urine testing may give a realistic estimate of overall adherence and is not necessarily affected prior to clinic visits due to a "white coat effect".

## Conclusion

Commercially available test strips use the same reaction but are expensive and difficult to acquire, while in-house prepared strips are easily produced and at very little cost. As the procedure is readily reproduced, thousands of strips could be routinely made to meet the testing needs of a given clinic population without significant time or cost.

Monitoring and enhancement of adherence should be part of TB treatment and control programs. In-house prepared urine test strips are inexpensive, effective and rapid, and could be used as a major evaluative tool in TB control programs.

## Authors' contributions

TS led the design, on-site implementation, and drafting of the manuscript. PK and JM helped in study conception, design, and institutional facilitation. AM and WC were involved in study conception, design, and data analysis. DW participated in study design, analysis and on-site supervision. MC performed the statistical analysis. All authors contributed to drafting of the manuscript. All authors read and approved the final manuscript.

## Declaration of competing interests

The author(s) declare that they have no competing interests.

## Pre-publication history

The pre-publication history for this paper can be accessed here:



## Supplementary Material

Additional File 1 Time-based urine result among volunteers taking a single-dose of INH 300 mg. Provides the results among 5 volunteers taking INH 300 mg once with urine testing at set intervals thereafter. Click here for file

Additional File 2 Responses to questionnaire overall and by hospital. Lists all of the questions of the questionnaire with the corresponding proportion of responses, separated based on hospital site. Click here for file

Additional File 3 Questionnaire. Copy of the actual questionnaire used in the study. Click here for file
