# Supplementary material for: Adherence with isoniazid for prevention of tuberculosis among HIV-infected adults in South Africa
Source: BMC Infect Dis. 2006 Jun 13;6:97. doi: 10.1186/1471-2334-6-97 (PMC1513236; doi:10.1186/1471-2334-6-97)
Supplement: Additional File 1 — Time-based urine result among volunteers taking a single-dose of INH 300 mg. Provides the results among 5 volunteers taking INH 300 mg once with urine testing at set intervals thereafter. [file 1471-2334-6-97-S1.doc]

| **Time-based urine result among volunteers taking a single-dose of INH 300 mg** | | | | |
| --- | --- | --- | --- | --- |
| Volunteer | **Urine result** | | | |
|  | ***Baseline*** | ***24 hours*** | ***36 hours*** | ***72 hours*** |
| 1 | - | + | + | - |
| 2 | - | + | + | - |
| 3 | - | + | + | - |
| 4 | - | + | + | - |
| 5 | - | + | - | - |
