# Supplementary material for: Adherence with isoniazid for prevention of tuberculosis among HIV-infected adults in South Africa
Source: BMC Infect Dis. 2006 Jun 13;6:97. doi: 10.1186/1471-2334-6-97 (PMC1513236; doi:10.1186/1471-2334-6-97)
Supplement: Additional File 2 — Responses to questionnaire overall and by hospital. Lists all of the questions of the questionnaire with the corresponding proportion of responses, separated based on hospital site. [file 1471-2334-6-97-S2.doc]

| **Responses to questionnaire overall and by hospital** | | | | | | |
| --- | --- | --- | --- | --- | --- | --- |
| **Variable** | **Hospital A**  **(n=99)** | | **Hospital B**  **(n=202)** | | **Overall**  **(n=302)** | |
|  | **n** | **%** | **n** | **%** | **n** | **%** |
| Urine result |  |  |  |  |  |  |
| Positive | 68 | 69.4 | 147 | 73.1 | 216 | 72.0 |
| Negative | 30 | 30.6 | 54 | 26.9 | 84 | 28.0 |
| Sex |  |  |  |  |  |  |
| Male | 19 | 19.2 | 50 | 24.9 | 69 | 22.9 |
| Female | 80 | 80.8 | 151 | 75.1 | 231 | 76.7 |
| Currently employed? |  |  |  |  |  |  |
| Yes | 27 | 27.3 | 38 | 18.8 | 65 | 21.6 |
| No | 72 | 72.7 | 164 | 81.2 | 236 | 78.4 |
| Years of education |  |  |  |  |  |  |
| 0-5 | 19 | 19.2 | 53 | 26.2 | 73 | 24.2 |
| 6-8 | 22 | 22.2 | 62 | 30.7 | 84 | 27.8 |
| 9-11 | 29 | 29.3 | 44 | 21.8 | 73 | 24.2 |
| 12+ | 29 | 29.3 | 43 | 21.3 | 72 | 23.8 |
| Transportation to Clinic† |  |  |  |  |  |  |
| Sector taxi | 87 | 87.9 | 138 | 68.3 | 225 | 74.8 |
| Walking | 3 | 3.0 | 37 | 18.3 | 40 | 13.3 |
| Car | 8 | 8.1 | 12 | 5.9 | 20 | 6.6 |
| Bus | 1 | 1.0 | 8 | 4.0 | 9 | 3.0 |
| Other | 0 | 0.0 | 7 | 3.5 | 7 | 2.3 |
| Time to get to clinic |  |  |  |  |  |  |
| < 1 hour | 55 | 55.6 | 124 | 61.4 | 179 | 59.7 |
| 1-24 hours | 44 | 44.4 | 75 | 37.1 | 119 | 39.7 |
| > 1 day | 0 | 0 | 2 | 1.0 | 2 | 0.6 |
| Have you told anyone you are taking INH?* |  |  |  |  |  |  |
| Yes | 70 | 71.4 | 167 | 83.1 | 238 | 79.3 |
| No | 28 | 28.6 | 34 | 16.9 | 62 | 20.7 |
| Have you told anyone you have HIV? |  |  |  |  |  |  |
| Yes | 76 | 77.6 | 171 | 85.1 | 248 | 82.7 |
| No | 22 | 22.4 | 30 | 14.9 | 52 | 17.3 |
| Taking anti-retrovirals? † |  |  |  |  |  |  |
| Yes | 12 | 12.2 | 6 | 3.0 | 18 | 6.0 |
| No | 86 | 87.8 | 194 | 97.0 | 281 | 94.0 |
| Are you taking the INH? |  |  |  |  |  |  |
| Yes | 97 | 100 | 201 | 100 | 299 | 100 |
| No | 0 | 0 | 0 | 0 | 0 | 0 |
| How frequently? |  |  |  |  |  |  |
| Every day | 89 | 90.8 | 196 | 97.5 | 286 | 95.3 |
| ≤ 6 days/week | 9 | 9.2 | 5 | 2.5 | 14 | 4.7 |
| Any side effects from INH? |  |  |  |  |  |  |
| Yes | 6 | 6.1 | 12 | 6.0 | 19 | 6.3 |
| No | 93 | 93.9 | 189 | 94.0 | 282 | 93.7 |
| Do you ever forget to take INH? † |  |  |  |  |  |  |
| Yes | 21 | 21.2 | 20 | 10.0 | 41 | 13.6 |
| No | 78 | 78.8 | 181 | 90.0 | 260 | 86.4 |
| Are you ever not sure when to take INH? |  |  |  |  |  |  |
| Yes | 10 | 10.1 | 9 | 4.5 | 19 | 6.3 |
| No | 89 | 89.9 | 192 | 95.5 | 282 | 93.7 |
| Do you know why you’re taking INH? |  |  |  |  |  |  |
| Yes | 36 | 36.7 | 63 | 31.2 | 99 | 33.0 |
| No | 62 | 63.3 | 139 | 68.8 | 201 | 67.0 |
| Has doctor/nurse explained why you’re taking INH? |  |  |  |  |  |  |
| Yes | 56 | 57.1 | 95 | 47.0 | 151 | 50.3 |
| No | 42 | 42.9 | 107 | 53.0 | 149 | 49.7 |
| Statement:: INH is dangerous to your health† |  |  |  |  |  |  |
| Strongly agree | 0 | 0.0 | 5 | 2.5 | 5 | 1.7 |
| Agree | 22 | 22.4 | 4 | 2.0 | 26 | 8.7 |
| Disagree | 61 | 62.2 | 23 | 11.5 | 84 | 28.1 |
| Strongly disagree | 2 | 2.0 | 129 | 64.5 | 132 | 44.1 |
| Don’t know | 13 | 13.3 | 39 | 19.5 | 52 | 17.4 |
| Statement:: Without INH, you’re chance of getting sick from TB is: † |  |  |  |  |  |  |
| High | 84 | 84.8 | 132 | 65.7 | 217 | 72.1 |
| Above average | 2 | 2.0 | 1 | 0.5 | 3 | 1.0 |
| Average | 5 | 5.1 | 3 | 1.5 | 8 | 2.7 |
| Below average | 0 | 0.0 | 1 | 0.5 | 1 | 0.3 |
| Don’t know | 8 | 8.1 | 64 | 31.8 | 72 | 23.9 |
| Do you ever not take INH because don’t know why should? |  |  |  |  |  |  |
| Yes | 0 | 0.0 | 3 | 1.5 | 3 | 1.0 |
| No | 99 | 100.0 | 198 | 98.5 | 298 | 99.0 |
| Which pharmacy supplies your INH† |  |  |  |  |  |  |
| Edendale | 0 | 0.0 | 114 | 56.7 | 114 | 37.9 |
| Grey’s | 95 | 96.0 | 1 | 0.5 | 96 | 31.9 |
| Peripheral | 4 | 4.0 | 86 | 42.8 | 91 | 30.2 |
| Are you ever unable to afford to go to pharmacy? † |  |  |  |  |  |  |
| Yes | 41 | 41.8 | 4 | 2.0 | 45 | 15.0 |
| No | 57 | 58.2 | 197 | 98.0 | 255 | 85.0 |
| Have you ever run out of INH between visits? † |  |  |  |  |  |  |
| Yes | 7 | 7.1 | 46 | 22.9 | 53 | 17.6 |
| No | 92 | 92.9 | 155 | 77.1 | 248 | 82.4 |
| Has your pharmacy ever run out of INH? † |  |  |  |  |  |  |
| Yes | 0 | 0.0 | 33 | 16.4 | 33 | 11.0 |
| No | 99 | 100.0 | 168 | 83.6 | 268 | 89.0 |
| There were 202 subjects from Hospital A, and 99 from Hospital B (Hospital not indicated on one out of 302 questionnaires)  * Hospital-specific proportions are significantly different at p < 0.05 level  † Hospital-specific proportions are significantly different at p < 0.01 level | | | | | | |
