# Supplementary material for: Adherence with isoniazid for prevention of tuberculosis among HIV-infected adults in South Africa
Source: BMC Infect Dis. 2006 Jun 13;6:97. doi: 10.1186/1471-2334-6-97 (PMC1513236; doi:10.1186/1471-2334-6-97)
Supplement: Additional File 3 — Questionnaire. Copy of the actual questionnaire used in the study. [file 1471-2334-6-97-S3.doc]

# Date (dd/mm/yy)____________________

Identifier # _________ Patient encounter site: □ Edendale □ Grey’s

**Isoniazid (INH) Questionnaire**

Please fill out the following questions. If you have any questions, a nurse

will be available to help you. Please check the response or fill in the blank next to each question. Thank you for your participation.

1. Age:_______ Sex: □ M □ F

2. Including yourself, how many people live in your household?_____________

3. Finances:

Are you currently employed? □ Yes □ No

# Including yourself, how many people are employed in your household?_________

# Including yourself, how many people are receiving grants in your household?_______

4. Please estimate your monthly household income: R___________/month

1. What level of education have you completed?

□ Standard (0-12) please specify_________ □ > 12

1. How do you get to this clinic:

□ Walk □ Bicycle □ Bus □ Car □ Taxi □ Other_____________

7. How much time does it take you to get to this clinic?

Check appropriate box and specify length:

□ Days____ □ Hours_____ □ Minutes______

1. Please describe why you are supposed to take the INH pills.

□ Yes □ No

9. Has your doctor/nurse explained to you the reasons for taking the isoniazid?

□ Yes □ No

10. Statement- The INH may be dangerous to my health (please check response):

□ strongly agree □ agree □ disagree □ strongly disagree □ don’t know

11. Statement- Without medication, my chance of getting sick from TB is:

□ high □ above average □ average □ below average □ don’t know

12. Have you told anybody that you are taking INH?

□ Yes □ No If yes, □ 1-10 (please specify)_______ □ > 10

13. Have you told anybody that you have HIV?

□ Yes □ No If yes, □ 1-10 (please specify)_______ □ > 10

1. Where do you get your medication most of the time?

□ Edendale □ Grey’s □ Peripheral clinic (name)____________

1. Have you been taking your INH pills?

□ Yes □ No

If yes, how often do you take them? When did you take the last dose?

□ Every day

□ 5 - 6 days per week □ ___________hours ago

□ 3 - 4 days per week

□ < 3 days per week □ I don’t remember

16. Please respond to whether the following statements have occurred to you while taking the INH pills:

|  | Yes | No |
| --- | --- | --- |
| You forget to take the medication |  |  |
| You are not sure when to take the medication |  |  |
| You can’t afford to go to the clinic to get my medications |  |  |
| You have side effects from the medication: |  |  |
| You run out of medication because your next appointment is made too late |  |  |
| The clinic has run out of your medication |  |  |
| You don’t take the pills because you don’t know why you’re supposed to |  |  |
| Other reasons: |  |  |

**Office use** (*you do not need to answer the questions in this part of the form*):

Date of initiation of INH therapy (dd/mm/yy)_____________

Time since last seen in CDC (dd/mm/yy)_______________

Date of HIV diagnosis (dd/mm/yy)___________________

Is the patient receiving any anti-retroviral medications?

□ Yes □ No

If yes, please list which:______________________________________

Urine: □ positive □ negative
